# Lipoprotein Combine Index Is Associated with Multi-Compartment Oxidative Stress in Clinically Stable Peritoneal Dialysis Patients: A Cross-Sectional Study

**DOI:** 10.3390/biomedicines14020456

**Published:** 2026-02-18

**Authors:** Natalia Stepanova, Lesya Korol

**Affiliations:** State Institution “O.O. Shalimov National Scientific Center of Surgery and Transplantology of the National Academy of Medical Science of Ukraine”, 03126 Kyiv, Ukraine; lesyakorol@meta.ua

**Keywords:** peritoneal dialysis, dyslipidemias, oxidative stress, antioxidants, peritoneal membrane, dialysis adequacy

## Abstract

**Background/Objectives**: Background: Dyslipidaemia and oxidative stress (OS) are frequent in peritoneal dialysis (PD). The Lipoprotein Combine Index (LCI) integrates lipid parameters, but its relationship with peritoneal transport and OS is unclear. **Methods:** This cross-sectional study included 100 clinically stable adults on continuous ambulatory PD with preserved ultrafiltration and adequate dialysis. LCI was calculated as (total cholesterol × triglycerides × LDL-C)/HDL-C and analyzed by tertiles. Lipid peroxidation and antioxidant markers were measured in serum, erythrocytes, urine, and spent dialysate. Multivariable regression models examined associations between LCI, peritoneal solute transport, and dialysate OS markers. **Results:** Higher LCI was independently associated with lower peritoneal solute transport. LCI correlated inversely with the 4 h dialysate-to-plasma creatinine ratio (ρ = −0.32, *p* = 0.001) and remained significant after adjustment (adjusted R^2^ = 0.224, *p* < 0.001). Increasing LCI was associated with higher malondialdehyde levels in serum, urine, and dialysate (all *p* ≤ 0.008) and impaired antioxidant defenses, including lower total peroxidase activity in erythrocytes and dialysate (both *p* = 0.001), reduced serum sulfhydryl groups (*p* = 0.011), decreased oxidative resistance of erythrocytes, and increased peroxide-induced hemolysis (both *p* = 0.001). In adjusted models, logLCI was independently associated with higher dialysate malondialdehyde (*p* < 0.001) and lower dialysate peroxidase activity (*p* = 0.005). **Conclusions:** In clinically stable PD patients, higher lipid burden assessed by LCI is independently associated with lower peritoneal solute transport and a marked increase in systemic and local OS. Our findings suggest that dyslipidaemia may contribute to early metabolic and oxidative changes even before overt peritoneal membrane dysfunction develops.

## 1. Introduction

Dyslipidaemia is common in patients undergoing peritoneal dialysis (PD) and has been widely studied as a contributor to high cardiovascular risk and patients’ mortality [1,2]. However, individual lipid fractions often present discordant patterns and their associations with dialysis and patient outcomes are inconsistent [2,3]. Therefore, single lipid parameters may not reflect the overall metabolic burden in PD.

The lipoprotein combine index (LCI) is a relatively new clinical tool that combines commonly used lipid profile markers into a single composite measure. It has effectively demonstrated cumulative lipid-related risk in different clinical contexts [4,5]. However, there is a general lack of data on LCI usage in PD patients.

Lipid peroxidation is a central component of OS, as circulating lipids serve as substrates for reactive oxygen species-mediated oxidative injury [6,7]. However, OS in patients receiving PD has only been the subject of a small number of original investigations [8,9], and few studies have addressed OS in the context of dyslipidaemia in this population; most have focused on its associations with dialysis adequacy or peritoneal clearance. Several studies have reported higher systemic or peritoneal OS markers in patients with lower dialysis dose or poorer technique outcomes [10,11,12,13]. These findings suggest that OS may reflect cumulative dialysate exposure and uremic burden.

At the same time, the link between OS and peritoneal transport status is not consistent across studies. Systemic markers of OS do not show a clear or uniform relationship with peritoneal transport status [12], suggesting that systemic and local oxidative processes may not always parallel each other. Conventional glucose-based PD solutions are well known to contribute to OS because of high glucose exposure and the presence of glucose degradation products [8,9]. However, oxidative and inflammatory activity is frequently decreased but not entirely eliminated even with glucose-sparing or more biocompatible dialysis regimens [14], suggesting that additional metabolic and uremia-related factors may play a role in driving OS in PD patients.

Despite these observations, few studies have examined lipid burden and OS across multiple biological compartments within the same framework. In our previous research, dyslipidaemia and OS were shown to be associated with impaired peritoneal ultrafiltration (UF), highlighting their clinical relevance [11]. However, those findings were primarily linked to overt functional impairment and did not clarify how lipid burden relates to peritoneal transport characteristics and OS in clinically stable patients with preserved UF and adequate dialysis delivery. Therefore, the present study aimed to examine the association between LCI, peritoneal transport status, and a multi-compartment OS profile in clinically stable PD patients with preserved UF.

## 2. Materials and Methods

### 2.1. Study Design and Ethical Approval

This study is a cross-sectional analysis derived from a prospective observational cohort originally designed to investigate dyslipidaemia and extracardiac risk in patients undergoing PD. The parent study was registered under the Domestic Trial Registration Number 0117U002122. The study protocol was reviewed and approved by the Ethics Committee of the State Institution “Institute of Nephrology of the National Academy of Medical Sciences,” Kyiv, Ukraine (Protocol No. 7, dated 12 September 2016). All study procedures were conducted in accordance with the Declaration of Helsinki. Written informed consent was obtained from all participants before enrollment.

### 2.2. Study Setting and Participants

Participants were recruited from three dialysis centers in Ukraine between January 2019 and December 2021. Each center used the same standardized baseline assessment protocol, which included the collection of clinical history, routine laboratory tests, peritoneal equilibration testing (PET), and measurements of lipid and OS markers.

Adults aged ≥ 18 years were eligible if they had been receiving continuous ambulatory PD (CAPD) for at least three months, were clinically stable at the time of assessment, had peritoneal UF of more than 400 mL per day, and achieved a total weekly Kt/V ≥ 1.7.

Patients were excluded if they had an acute intercurrent illness, active infection, or peritonitis within the previous four weeks. Additional exclusion criteria were prior hemodialysis treatment, recent myocardial infarction or decompensated heart failure (NYHA class III-IV) within the past six months, known chronic liver disease or active hepatitis, active chronic inflammatory or autoimmune conditions, major surgery or significant trauma within the previous three months, and any known malignancy. Patients with anuria (24 h urine output < 100 mL) and those using icodextrin or other glucose-free PD solutions were also excluded.

For this study, “clinically stable” was defined as the absence of acute medical events, hemodynamic instability, recent hospitalization, significant changes in PD prescription, or major alterations in clinical status or medication regimen within the preceding four weeks.

CAPD was performed using Dianeal PD4 solutions with glucose concentrations of 1.36% and 2.27% in 2.0 L Twin Bag systems (Baxter Healthcare Corporation, Deerfield, IL, USA).

### 2.3. Sample Size

No formal a priori sample size calculation was performed for this study. The final sample size was determined by the number of eligible patients with complete baseline data available during the study period.

Post hoc power analysis was conducted in G*Power (version 3.1.9.7; Heinrich-Heine-Universität Düsseldorf, Düsseldorf, Germany) using the exact test for correlation (bivariate normal model; two-tailed α = 0.05). With a total sample size of 100, the achieved power to detect a medium correlation effect size (Cohen’s r = 0.30) was approximately 0.87. Based on the observed association between log-transformed LCI and dialysate-to-plasma creatinine ratio (D/P Cr) (Spearman ρ = −0.32), the corresponding achieved power was approximately 0.91.

For between-group comparisons across three LCI tertiles, power was approximated using a one-way ANOVA omnibus test (as an approximation to the Kruskal–Wallis design; two-sided α = 0.05, total *n* = 100). Under this approximation, the achieved power was approximately 0.88 for a moderate-to-large effect size (Cohen’s f = 0.35). The final sample size of 100 patients was consistent with that reported in similar studies examining oxidative stress and peritoneal transport in peritoneal dialysis populations [12,13].

### 2.4. Baseline Data Collection and Study Procedures

Baseline demographic and clinical data, laboratory sampling, and dialysis adequacy assessments were performed according to a standardized protocol previously described in detail by our group [15].

Briefly, routine biochemical parameters, such as urea, creatinine, albumin, total protein, C-reactive protein, glucose, electrolytes, intact parathyroid hormone, hemoglobin (Hb), and lipid profile, were measured at the initial study visit following an overnight fast. Patients collected 24 h urine and spent dialysate the day before this appointment to measure urea, creatinine, and total volume.

A standard PET was performed 1–2 days later, following the protocol of Twardowski et al. [16]. At the start of the PET, before the instillation of fresh dialysate, fasting blood samples were obtained for OS analyses. Dialysate samples for OS assessment were collected at the end of the 4 h dwell. Serum and dialysate samples were processed immediately.

Dialysis adequacy and peritoneal transport status were assessed as previously described [15]. In brief, total weekly urea clearance (Kt/V) and creatinine clearance (CrCl) were calculated; peritoneal and renal Kt/V were estimated separately. Residual kidney function was assessed using 24 h urine volume and weekly renal Kt/V. Peritoneal transport status was characterized by the 4 h D/P Cr obtained from the PET.

Daily peritoneal glucose load was estimated based on the number and concentration of dialysate exchanges, assuming 27.2 g of glucose per 2.0 L 1.36% bag and 45.4 g per 2.0 L 2.27% bag. Total glucose load (g/day) was calculated as the sum of glucose contained in all exchanges performed per day.

The lipid profile included triglycerides (TGs), total cholesterol (TC), high-density lipoprotein cholesterol (HDL-C), low-density lipoprotein cholesterol (LDL-C), and very low-density lipoprotein cholesterol (VLDL-C). Log (TG/HDL-C) was used to compute the atherogenic index of plasma (AIP).

An automated Flexor Junior analyzer (Vital Scientific, Spankeren, The Netherlands) was used for biochemical assays, and an ABX Micros-60 analyzer (Horiba Medical, Montpellier, France) was used to measure hematological parameters.

### 2.5. LCI Calculation

LCI was derived as: LCI = (TC × TG × LDL-C)/HDL-C (all mmol/L). For subsequent analyses, patients were stratified into tertiles based on LCI values (T1: lowest tertile, T2: middle tertile, and T3: highest tertile).

### 2.6. OS Measurements

To evaluate systemic and local redox status in PD patients, markers of lipid peroxidation and antioxidant defense were assessed in serum, spent dialysate, erythrocytes, and urine using methods previously reported by our group [11,17].

Lipid peroxidation was estimated by determining malondialdehyde levels in serum (MDAs), dialysate (MDAd), erythrocytes (MDAe), and urine (MDAu). Venous blood samples were centrifuged to separate serum and erythrocyte fractions for the determination of MDAs and MDAe. The samples were treated with 17% trichloroacetic acid, after which centrifuged, and then the supernatant was reacted with 0.8% thiobarbituric acid under boiling conditions, with absorbance being measured at 532 nm. Concentrations were expressed as µmol/L for serum, dialysate, and urine, and as nmol/g Hb for erythrocytes.

For the antioxidant and redox-related characterization, we assessed the serum ceruloplasmin, total peroxidase activity in erythrocytes (TPAe) and dialysate (TPAd), sulfhydryl groups in serum (SHs groups) and erythrocytes (SHe groups), as well as erythrocyte peroxide resistance and peroxide-induced hemolysis.

The ceruloplasmin concentration was measured by mixing 0.05 mL of serum with 4 mL of 0.4 M acetic buffer (pH 5.5) and 0.5 mL of a 0.5% aqueous solution of 1,2-phenylenediamine dihydrochloride. Absorbance was recorded at 530 nm, and results were expressed in g/L.

For the TPAe determination, 0.5 mL of erythrocyte hemolysate (diluted 1:1000) was mixed with 1 mL of 0.2 M acetate buffer (pH 4.9) and 1 mL of 0.05 mM Indigo Carmine solution, afterwards incubated at 30 °C for 5 min. The reaction was initiated by adding 0.5 mL of 0.03 M hydrogen peroxide solution to the test samples, with the control samples receiving 0.5 mL of distilled water. After 2 min, the reaction was stopped by adding 3 mL of 20% sulfuric acid. Absorbance was measured at 670 nm, and TPA activity was expressed as U/g.

TPAd was determined using the same spectrophotometric principle as for erythrocytes, with minor modification for the aqueous matrix of dialysate. In short, 0.5 mL of dialysate was combined with 1 mL of 0.2 M acetate buffer (pH 4.9) and 1 mL of 0.05 mM Indigo Carmine solution and incubated at 30 °C for 5 min. The reaction was initiated by adding 0.5 mL of 0.03 M hydrogen peroxide solution; control samples received 0.5 mL of distilled water. After 2 min, 3 mL of 20% sulfuric acid was added to terminate the reaction. Absorbance was measured at 670 nm, and TPA activity in dialysate was expressed as U/L.

The concentration of SHs groups was evaluated by diluting 0.05 mL of serum in 0.5 mL of distilled water, followed by the addition of potassium iodide solution, starch solution, and phosphate buffer. Absorbance was measured both before and after iodine was added, and SH group concentration was expressed in mmol/L.

For erythrocytes, SH groups were determined in washed erythrocyte hemolysate prepared from the same blood sample. In short, using the same spectrophotometric method as for serum, 0.05 mL of erythrocyte hemolysate was diluted to 0.5 mL with distilled water, and then potassium iodide solution, starch solution, and phosphate buffer were added. The concentration of the SHe group was expressed as mmol/L, and absorbance was measured both before and after the iodine solution was added.

Two functional tests, peroxidase resistance and peroxide-induced hemolysis, were also carried out on washed erythrocyte suspensions in order to coordinate the evaluation of erythrocyte resistance to oxidative damage and membrane stability under pro-oxidant circumstances.

Peroxidase resistance (%) was determined as a measure of the ability of erythrocytes to withstand oxidative challenge. This test reflects the functional capacity of erythrocyte antioxidant defense systems to counteract reactive oxygen species.

Peroxide-induced hemolysis (%) was evaluated as an indicator of erythrocyte membrane vulnerability to oxidative damage. The extent of hemolysis after exposure to hydrogen peroxide was used to characterize the susceptibility of red blood cell membranes to peroxidative injury.

All the reagents used in the assays were only of analytical grade and were obtained from commercial suppliers. Tris(hydroxymethyl)aminomethane, tris(hydroxymethyl)aminomethane hydrochloride, malonaldehyde bis(diethyl acetal), 1,4-phenylenediamine dihydrochloride, human ceruloplasmin, sodium fluoride, potassium chloride, and potassium iodide were purchased from Sigma-Aldrich ((St. Louis, MO, USA). Trichloroacetic acid, thiobarbituric acid, ferric ammonium citrate, sodium hydrogen phosphate, and sodium acetate, on the other hand, were obtained from Merck KGaA (Darmstadt, Germany). Every test was run twice, and the samples were blinded to the clinical data during analysis.

### 2.7. Statistical Analysis

All statistical analyses were performed using Jamovi (version 2.3.21, Sydney, Australia) and MedCalc (version 22.006, Ostend, Belgium). Continuous variables were presented as median (Me) and interquartile range (Q25–Q75) or mean (M) ± standard deviation (SD) based on the Shapiro–Wilk test. Comparisons between the three LCI tertiles were performed using one-way ANOVA for normally distributed variables or the Kruskal–Wallis test for non-normally distributed variables, as appropriate. When the overall test was significant, Dunn’s post hoc pairwise comparisons were conducted with correction for multiple testing. Categorical variables were compared using the Chi-square test (χ^2^). Bivariate associations assessed by Spearman’s rank correlation (ρ).

Multivariable linear regression models were constructed to examine the associations between LCI (exposure) and (1) the D/P Cr ratio and (2) dialysate OS markers (MDAd and TPAd), which were treated as dependent variables. Because of the relatively small sample size in the dialysate subset (n = 48), covariate adjustment was limited to a parsimonious set of biologically relevant confounders (age, sex, diabetes status, and estimated daily glucose load). Natural logarithmic transformation was applied to LCI and dialysate OS markers (logLCI, logMDAd, and logTPAd) because of skewed distributions. Sensitivity analyses were performed by additionally adjusting the dialysate OS models for logCRP, serum albumin, and statin therapy. In the peritoneal transport models (full cohort), these covariates were incorporated in a fully adjusted model. Analyses were performed using complete-case data within each model; dialysate OS markers were available in a subset of patients. No imputation was required, as all variables included in each model were complete for the analyzed cases. Hypothesis-driven comparisons were prespecified, and no formal correction for multiple testing was applied.

## 3. Results

### 3.1. Cohort Characteristics

**Figure 1:**
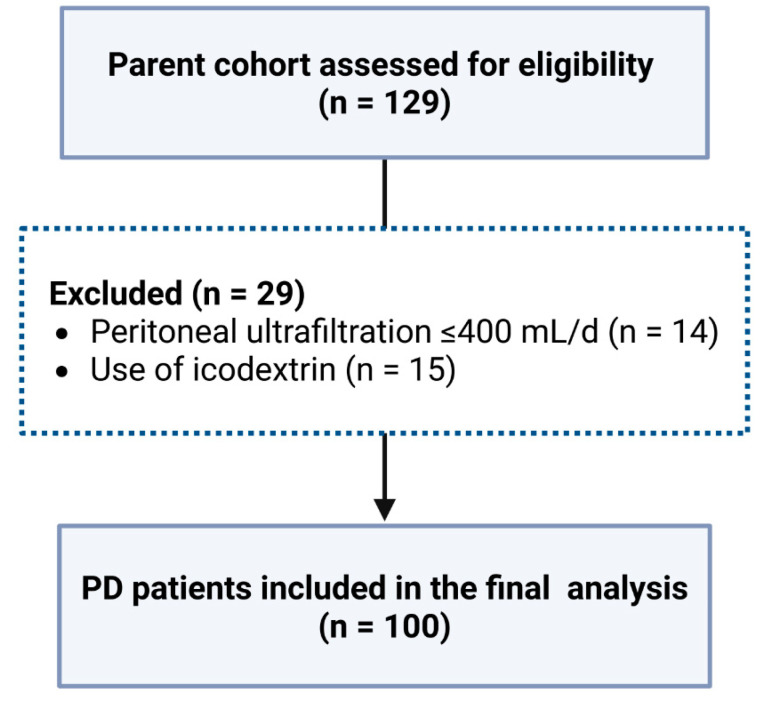
Flow Diagram of Patient Selection for the Present Analysis.

Of the 129 patients in the parent cohort, 100 met all inclusion criteria and were included in the final analysis (Figure 1).

LCI values showed substantial variability among patients, ranging from 3.6 to 225.9, with a median of 23.7 (11.4–61.3). Baseline demographic, clinical, and PD-related characteristics of the included patients stratified by LCI tertiles are presented in Table 1.

As presented in Table 1, diabetes was more common in patients in the highest LCI group (T3). These patients also had higher systolic and diastolic blood pressure compared with those in T1 and T2. Blood albumin levels varied between the groups and were lower in T3 than in T2, while blood calcium levels were highest in the T3 group.

As expected, atherogenic lipid markers increased progressively across LCI tertiles. Although HDL-C did not differ significantly between the groups, TC, LDL-C, VLDL-C, triglycerides, and AIP were significantly higher in T2 and T3 compared with the T1 group.

In addition, patients in the highest LCI tertile exhibited a significantly lower 4 h D/P Cr ratio and a higher prevalence of low-average transport status compared with lower tertiles. The estimated daily glucose load was significantly higher in the T3 group compared with both T1 and T2. Among medications used, statin therapy was significantly more common in patients in the T2 and T3 LCI tertiles compared with the T1 group. All other demographic, clinical, laboratory, and dialysis-related parameters were similar across LCI tertiles.

### 3.2. Association Between LCI and Peritoneal Transport Characteristics

Beyond the descriptive differences across tertiles presented in Table 1, we examined the continuous relationship between LCI and peritoneal transport status.

**Figure 2:**
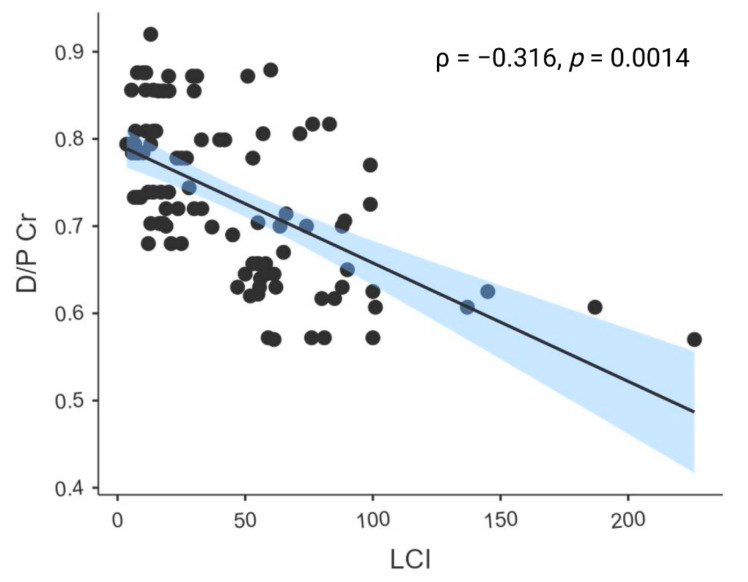
Association Between LCI and Peritoneal Transport Status in PD Patients. Spearman’s rank correlation coefficient (ρ) was calculated. The solid line represents the linear regression fit, and the shaded area indicates the 95% confidence interval.

In bivariate analysis, LCI was inversely correlated with the 4 h D/P Cr ratio, indicating that lower peritoneal solute transport was associated with higher LCI values (Figure 2).

**Figure 3:**
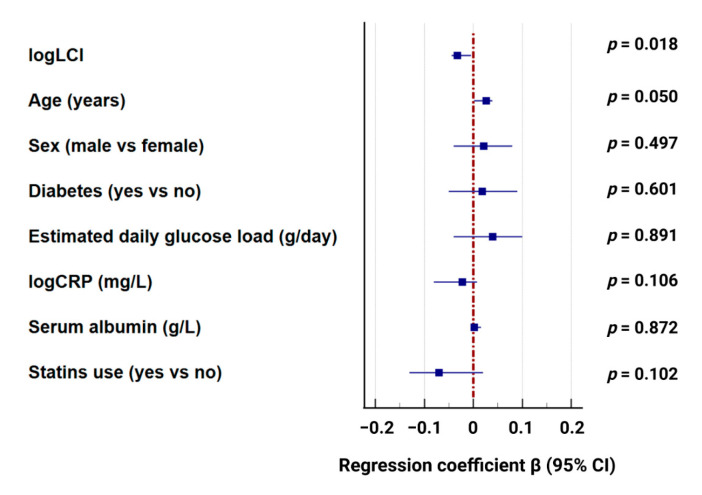
Multivariable Linear Regression Analysis of Factors Associated with D/P Cr ratio.

To assess whether this association was independent of other potential confounders, multivariable linear regression was performed with the 4 h D/P creatinine ratio as the dependent variable and log-transformed LCI (logLCI) as the main independent variable. After adjustment for age, sex, diabetes status, estimated daily glucose load, CRP, serum albumin, and statin use, higher logLCI remained significantly associated with lower D/P Cr. The overall model was statistically significant (R^2^ = 0.230, adjusted R^2^ = 0.124; F = 2.17, *p* = 0.024) (Figure 3).

### 3.3. LCI and OS Markers

Across LCI tertiles, significant differences were observed in several markers of lipid peroxidation and antioxidant defense, indicating a progressive shift toward a more pro-oxidant profile with increasing LCI. MDAs, MDAu, and MDAd were markedly higher in the LCI T3 group, suggesting greater systemic and peritoneal lipid peroxidation in patients with higher LCI (Table 2).

With respect to antioxidant capacity, TPA was lower in T3 compared with T2 in both erythrocytes and dialysate. SHs groups also differed across tertiles, whereas SHe showed no significant variation.

Functional assays of erythrocyte susceptibility to OS demonstrated significant differences across LCI tertiles: peroxide resistance was lower in T2 and T3 compared with T1, while peroxide-induced hemolysis was higher in T2 and T3, indicating increased membrane vulnerability to oxidative injury with higher LCI. Serum ceruloplasmin levels also differed across tertiles, with lower values in T3 compared with T2.

In multivariable linear regression models adjusted for age, sex, diabetes status, and estimated daily glucose load, logLCI was independently associated with both studied dialysate OS markers. For MDAd, the overall model was statistically significant (R^2^ = 0.62, adjusted R^2^ = 0.57; F = 11.2, *p* < 0.001). Higher logLCI was independently associated with higher logMDAd (Table 3).

Estimated glucose load and diabetes status were directly associated with dialysate MDAd, whereas age and sex were not significant predictors in the adjusted model.

For TPAd, in a separate adjusted model (R^2^ = 0.39, adjusted R^2^ = 0.33; F = 6.9, *p* < 0.001), higher logLCI was independently associated with lower logTPAd. Higher estimated glucose load and diabetes were also associated with lower TPAd, whereas male sex was linked to higher TPAd (see Table 3).

### 3.4. Sensitivity Analyses

To evaluate robustness, additional models were fitted with further adjustment for serum albumin, logCRP, and statin therapy.

For MDAd, the fully adjusted model remained statistically significant (R^2^ = 0.689, adjusted R^2^ = 0.608; F(8, 31) = 8.57, *p* < 0.001). Higher logLCI remained independently associated with higher logMDAd. Male sex, diabetes status, and estimated glucose load were positively associated with MDAd, whereas serum albumin was inversely associated. Age, statin therapy, and logCRP were not independently associated (Supplementary Table S1).

For TPAd, results were directionally consistent in fully adjusted models, with higher logLCI remaining independently associated with lower logTPAd. Estimated glucose load showed an inverse association, while male sex and statin therapy were positively associated. Diabetes status, serum albumin, and logCRP were not significant predictors (Supplementary Table S2).

Importantly, because of the limited sample size in the dialysate subset, these extended models included multiple covariates and should be interpreted cautiously. Nevertheless, the consistency of effect direction across primary and sensitivity analyses supports the robustness of the main findings.

## 4. Discussion

The present study is the first to examine the association between atherogenic lipid burden, assessed by LCI, transport status, and multi-compartment OS markers in clinically stable PD patients with preserved peritoneal UF and adequate dialysis delivery.

A key finding of this study is the independent inverse association between LCI and the 4 h D/P Cr ratio. Low-average transporters were highly overrepresented in the highest LCI tertile compared with the lower tertiles. This association remained robust after adjustment for age, sex, diabetes, daily glucose load, CRP, serum albumin, and statin therapy.

Our findings differ from earlier PD studies, including our previous work. In analyses of the parent cohort stratified by UF failure, a greater prevalence of dyslipidaemia was associated with higher peritoneal transport status [11]. Liu et al. showed that high transporters had greater peritoneal protein losses, lower serum albumin, and more severe malnutrition [18]. Chang et al. demonstrated that rapid transport was positively correlated with protein losses, whereas triglyceride levels were inversely associated with protein clearance [19]. In these cohorts, rapid transport was commonly associated with UF failure, inflammation, protein-energy wasting, and technique failure. Under these conditions, lipid abnormalities reflected advanced peritoneal membrane injury and excessive systemic glucose absorption. Recent evidence, however, challenges the assumption that peritoneal glucose absorption alone determines lipid abnormalities [20,21], suggesting that systemic glucose absorption per se may not be the primary driver of dyslipidaemia in PD.

In contrast, the present cohort represents a different clinical context. All patients were clinically stable, had preserved UF, and met adequacy targets. Despite this stability, patients in the highest LCI tertile had both lower serum albumin levels and higher prescribed daily glucose loads. With preserved UF, reduced albumin is unlikely to reflect excessive protein loss. Instead, it may reflect low-grade inflammation, OS, or metabolic dilution effects, in line with the observed impairment of antioxidant defenses and increased lipid peroxidation. Higher estimated glucose load in LCI T3 was required to maintain UF because of slower solute equilibration during long dwells. This led to prolonged intraperitoneal glucose exposure rather than rapid systemic glucose absorption. In this setting, lipid burden appears to reflect cumulative metabolic and OS rather than advanced membrane dysfunction. In addition, another study reported no significant association between conventional lipid parameters and peritoneal transport characteristics [22], showing some heterogeneity in the lipid-transport relationship among PD patients.

Importantly, peritoneal transport status is not static. Longitudinal studies have shown that transport characteristics may change over time and may partially reverse in some patients [23]. These changes suggest that transport status is a reflection of the balance between membrane structure, metabolic exposure, and local inflammatory processes. In this case, our results likely represent an early or intermediate stage of peritoneal membrane adaptation where lipid burden and OS are already increased despite preserved UF and adequate clearance.

Another important finding of this study is a strong association between LCI and OS markers across systemic and peritoneal compartments. PD patients with the highest LCI showed higher levels of MDA in serum, urine, and dialysate, indicating that oxidative injury was present both systemically and within the peritoneal cavity. Antioxidant capacity was simultaneously reduced, as indicated by lower TPA, variations in SH groups availability, decreased resistance of erythrocytes to an oxidative challenge, and increased peroxide-induced hemolysis. Taken together, these findings suggest that a higher lipid burden is associated with a significant shift toward a pro-oxidant state in patients treated with PD.

These results extend previous studies of systemic or peritoneal OS in PD, which primarily linked elevated OS to dialysis inadequacy, prolonged glucose exposure, and membrane dysfunction [12,24,25,26]. However, few have looked into the role of lipid burden in redox imbalance. The present study showed consistent relationships between the LCI and multi-compartment OS among clinically stable patients, thus adding another metabolic dimension to the understanding of OS in PD.

The findings were particularly interesting for dialysate OS markers. After accounting for age, sex, diabetes, and estimated daily glucose load, higher LCI remained independently associated with increased MDAd and reduced TPAd. The associations remained unchanged in sensitivity analyses, additionally adjusting for CRP, serum albumin, and statin therapy. This observation is noteworthy because it implies that lipid burden may contribute directly to local peritoneal OS, rather than acting solely through glucose-related mechanisms. Previous studies have identified dyslipidaemia as a meaningful factor in peritoneal membrane dysfunction and reduced technique survival [27,28,29]. In this context, the observed relationship between LCI and peritoneal OS offers a plausible biological mechanism through which dyslipidaemia may adversely affect long-term peritoneal membrane integrity and, ultimately, technique outcomes in patients undergoing PD.

Biopsy-based studies of human peritoneal tissue further support the relevance of OS to structural peritoneal injury [30]. PD patients have been shown to exhibit increased oxidative DNA damage, indicated by higher 8-hydroxy-2′-deoxyguanosine levels in plasma and greater γH2AX expression in mesothelial cells and pericapillary walls compared with controls, with more pronounced changes in advanced peritoneal disease [30]. Together, these findings show that OS extends beyond circulating and dialysate compartments and also affects the cellular and vascular structures of the peritoneal membrane, providing a structural basis for the OS patterns observed in the present study.

The relationship between estimated glucose load, diabetes, and OS in the study context deserves particular attention. Although a higher daily glucose load was independently associated with increased MDAd and reduced TPAd, OS markers remained strongly associated with LCI after adjustment for glucose exposure. This finding suggests that glucose alone does not fully explain the oxidative burden observed in our cohort. Lipids may act as important substrates for oxidative reactions [31,32], providing targets for reactive oxygen species and thereby amplifying lipid peroxidation processes within both systemic circulation and the peritoneal cavity.

Diabetes emerged as another important modifier of both lipid burden and OS [33,34]. In line with the well-known pro-oxidant milieu linked to chronic hyperglycemia, patients with diabetes showed increased lipid peroxidation and decreased antioxidant activity [35,36]. Nonetheless, the association between LCI and OS in the models suggests that composite dyslipidemia reflects metabolic risk in addition to diabetes. These results corroborate the potential utility of LCI as an integrative marker of cumulative lipid-related oxidative burden in patients undergoing PD.

Several limitations of this study should be acknowledged. First, its cross-sectional design does not allow causal conclusions. The temporal relationship between lipid burden, peritoneal transport status, and OS cannot be determined. It remains unclear whether increased lipid burden contributes to OS and transport alterations or whether these processes evolve in parallel as part of broader metabolic changes in PD. Second, the sample size was moderate, and dialysate OS markers were available only in a subset of patients. This may have limited statistical power for some analyses. Although the observed associations were robust in multivariable models, larger cohorts are needed to confirm these findings and to allow more extensive adjustment for potential confounders. Third, only clinically stable patients using conventional glucose-based CAPD solutions were included. Patients with UF failure or those receiving biocompatible PD solutions were excluded. Although this criterion allowed assessment of early metabolic and oxidative changes in a stable setting, it limits the generalizability of the results to broader PD populations. Fourth, glucose load was estimated from prescribed dialysate concentrations rather than directly measured absorption. This may not fully reflect individual variability in glucose transfer. Fifth, OS was assessed using indirect biochemical markers. Although multiple compartments were examined, these measures may not capture all aspects of in vivo redox processes. Finally, residual confounding cannot be excluded. Factors such as dietary intake, physical activity, inflammation-related biomarkers, variability in medication, and genetic susceptibility were not assessed due to the sample size restriction and might have impacted lipid metabolism or OS.

## 5. Conclusions

In clinically stable PD patients with preserved UF and adequate dialysis delivery, higher LCI was associated with lower peritoneal solute transport and a more pronounced OS profile across systemic and peritoneal compartments. Our findings suggest that cumulative lipid burden may promote OS even in stable PD patients with low-average peritoneal transport status and may represent an early stage of metabolic and redox imbalance before obvious peritoneal membrane dysfunction occurs.

Further longitudinal studies are needed to clarify the clinical implications of these associations and determine whether lipid-lowering therapy may influence OS and long-term peritoneal membrane outcomes.

## Figures and Tables

**Table 1:** Patients’ Characteristics Stratified by LCI Tertiles.

Parameter	All Patients (n = 100)	LCI T1 (<16) (n = 34)	LCI T2 (17–45) (n = 32)	LCI T3 (>45) (n = 34)	*p*-Value
Demographic and clinical data					
Male gender, n (%)	48 (48.0%)	12 (35.3%)	18 (56.2%)	18 (52.9%)	0.182
Age, years	49.9 ± 12.6	48.4 ± 13.2	48.0 ± 10.1	53.2 ± 10.5	0.169
Diabetes, n (%)	36 (36.0%)	4 (11.8%) ^2,3^	12 (37.5%) ^1,3^	20 (58.8%) ^1,2^	0.0005
Systolic blood pressure, mm Hg	130 (110–140)	130 (110–140) ^3^	130 (120–140) ^3^	140 (130–160) ^1,2^	<0.0001
Diastolic blood pressure, mm Hg	90 (80–95)	90 (70–90) ^3^	90 (80–90) ^3^	100 (90–100) ^1,2^	<0.0001
BMI, kg/m^2^	24.5 (21.0–29.2)	23.7 (20.8–29.3)	24.8 (20.9–29.7)	24.5 (22.3–25.5)	0.795
Serum albumin, g/L	38.5 (34.4–40.8)	36.6 (32.8–39.5)	40.5 (38.1–41.7) ^3^	35.8 (34.4–40.5) ^2^	0.046
Total protein, g/L	66.1 (58.1–68.2)	63.6 (57.9–67.4)	66.3 (64.7–67.8)	64.8 (56.7–72.0)	0.311
CRP, mg/L	9.8 (5.5–18.7)	9.8 (4.3–18.5)	8.8 (6.7–17.2)	10.5 (6.1–20.7)	0.216
Hb, g/L	94.0 (92.5–109.5)	92.0 (90.5–119.3)	97.2 (92.0–111.6)	101.0 (93.1–107.7)	0.123
Glucose, mmol/L	5.5 (5.0–7.6)	5.08 (4.95–7.57)	5.50 (5.11–6.87)	5.62 (5.34–7.62)	0.783
Potassium, mmol/L	4.5 ± 1.07	4.2 ± 1.02	4.5 ± 1.09	4.7 ± 1.13	0.081
Calcium, mmol/L	2.34 (2.20–2.37)	2.26 (1.9–2.26) ^2,3^	2.34 (2.16–2.36) ^1^	2.36 (2.29–2.45) ^1^	0.0004
Phosphorus, mmol/L	2.0 (1.6–2.5)	2.1 (1.9–2.5)	1.8 (0.7–2.2)	1.9 (1.6–2.4)	0.067
iPTH, ng/L	206 (70.0–336.0)	206 (70–337)	227.5 (61.7–249)	231 (60–334)	0.215
Lipid profile markers					
LCI	23.7 (11.4–61.3)	7.8 (5.8–11.4) ^2,3^	23.7 (20.2–32.8) ^1,3^	71.4 (61.3–80.5) ^1,2^	<0.0001
Total cholesterol, mmol/L	5.6 (5.1–6.6)	4.5 (3.3–5.2) ^2,3^	6.2 (5.1–6.2) ^1,3^	6.7 (6.6–7.3) ^1,2^	<0.0001
HDL-C, mmol/L	1.14 (0.95–1.42)	1.14 (1.0–1.21)	1.42 (1.06–1.43)	1.08 (0.88–1.25)	0.081
LDL-C, mmol/L	4.15 (3.32–4.39)	3.25 (2.60–3.62) ^2,3^	4.22 (3.59–4.26) ^1,3^	4.45 (4.26–4.69) ^1,2^	<0.0001
VLDL-C, mmol/L	0.63 (0.42–1.16)	0.30 (0.24–0.35) ^2,3^	0.56 (0.49–0.66) ^1,3^	1.37 (1.08–1.71) ^1,2^	<0.0001
Triglycerides, mmol/L	1.44 (1.11–2.48)	0.82 (0.73–1.28) ^2,3^	1.27 (1.18–1.47) ^1,3^	2.81 (2.28–3.43) ^1,2^	<0.0001
AIP	4.91 (4.07–5.60)	3.63 (1.77–4.28) ^2,3^	5.23 (4.55–5.27) ^1,3^	5.61 (4.86–6.19) ^1,2^	<0.0001
PD-related parameters					
Time on PD, months	36 (26–49)	36 (30–58)	36 (19–44)	32 (26–62)	0.601
Urine volume, L/24 h	0.48 (0.25–0.80)	0.45 (0.25–0.80)	0.55 (0.30–0.75)	0.40 (0.20–0.90)	0.861
Peritoneal UF, mL/d	700 (550–900)	750 (600–900)	700 (500–900)	650 (500–1000)	0.415
Previous peritonitis episode, n (%)	57 (57.0%)	19 (55.9%)	17 (53.1%)	21 (61.7%)	0.483
4 h D/P Cr ratio	0.77 (0.70–0.82)	0.78 (0.73–0.81) ^3^	0.79 (0.71–0.86) ^3^	0.70 (0.64–0.81) ^1,2^	0.019
Low-average transporters, n (%)	16 (16.0%)	2 (5.9%) ^3^	2 (6.2%) ^3^	12 (35.3%) ^1,2^	0.004
High-average transporters, n (%)	56 (56.0%)	24 (70.6%) ^3^	18 (56.2%)	14 (41.2%) ^1^	0.016
High transporters. n (%)	28 (28.0%)	8 (23.5%)	12 (37.5%)	8 (23.5%)	0.221
Peritoneal weekly Kt/V	1.91 (1.38–2.31)	1.91 (1.46–2.23)	1.92 (1.88–2.13)	1.89 (1.33–2.39)	0.343
Renal weekly Kt/V	0.18 (0.12–0.62)	0.23 (0.14–0.39)	0.17 (0.12–0.44)	0.19 (0.11–0.81)	0.439
Total weekly Kt/V	2.05 (1.85–2.32)	2.03 (1.96–2.31)	2.14 (1.83–2.49)	2.00 (1.91–2.10)	0.581
Peritoneal weekly CrCl, L/week/1.73 m^2^	48.3 (43.2–52.6)	48.7 (45.2–53.3)	47.4 (42.6–53.7)	48.2 (41.6–51.5)	0.694
Estimated peritoneal glucose load, g/d	163.4 (145.2–181.6)	163.4 (163.4–163.4) ^3^	163.4 (145.2–172.5) ^3^	181.6 (145.2–227.0) ^1,2^	0.003
Medications					
ACE inhibitors/RAAS blockers, n (%)	82 (82.0%)	26 (76.4%)	27 (84.4%)	29 (85.3%)	0.360
Diuretics, n (%)	63 (63.0%)	22 (64.7%)	20 (62.5%)	21 (61.8%)	0.808
Iron supplementation, n (%)	65 (65.0%)	23 (67.6%)	24 (75.0%)	18 (52.9%)	0.064
Erythropoietins, n (%)	71 (84.5%)	25 (73.5%)	24 (75.0%)	22 (64.7%)	0.366
Non-calcium phosphate binders, n (%)	18 (18.0%)	6 (17.6%)	5 (15.6%)	7 (20.6%)	0.601
Statins, n (%)	44 (44.0%)	12 (35.3%) ^2,3^	4 (12.5%) ^1,3^	28 (82.4%) ^1,2^	<0.0001

Continuous variables are presented as mean ± SD or median (interquartile range, Q25–Q75), and categorical variables as number (%). Superscript numbers (^1–3^) indicate statistically significant pairwise differences between LCI tertiles based on Dunn’s post hoc test with correction for multiple comparisons: ^1^ significant vs. LCI T1; ^2^ significant vs. LCI T2; ^3^ significant vs. LCI T3. Abbreviations: ACE, angiotensin-converting enzyme; AIP, atherogenic index of plasma; BMI, body mass index; CrCl, creatinine clearance; CRP, C-Reactive Protein; D/P creatinine ratio, dialysate/plasma creatinine ratio; Hb, hemoglobin; HDL, high-density lipoproteins; iPTH, intact parathyroid hormone; LCI, lipoprotein combine index; LDL, low-density lipoproteins; total Kt/V, total weekly Kt/V urea; RAAS, renin–angiotensin–aldosterone system; VLDL, very-low-density lipoproteins; UF, ultrafiltration.

**Table 2:** OS markers in PD patients stratified by LCI tertiles.

	All Patients (n = 100)	LCI T1 (<16) (n = 34)	LCI T2 (17–45) (n = 32)	LCI T3 (>45) (n = 34)	*p*-Value
MDAs, µmol/L	474.3 (384.6–576.9)	397.4 (371.8–474.3) ^2,3^	499.9 (410.2–551.2) ^1,3^	564.1 (435.0–743.6) ^1,2^	**0.008**
MDAe, nmol/g Hb	602.5 (461.5–1038.4)	602.5 (551.2–1282.0)	506.4 (358.9–961.5)	846.2 (461.5–1038.0)	0.237
MDAu, µmol/L	448.7 (217.9–666.0)	262.8 (205.1–512.9) ^3^	294.9 (173.1–435.9) ^3^	679.50 (564.0–679.5) ^1,2^	**<0.0001**
MDAd, µmol/L	12.8 (10.5–19.6)	19.6 (12.7–19.6) ^3^	11.0 (0.05–12.7) ^3^	51.3 (12.9–51.3) ^1,2^	**<0.0001**
TPAe, U/g	623.1 (513.3–875.8)	630.6 (509.3–1108.0) ^2^	875.6 (585.5–1284.5) ^1,3^	546.50 (517.4–620.7) ^2^	**0.001**
TPAd,. U/L	28.6 (11.4–37.5)	32.0 (11.9–40.6) ^2,3^	26.3 (23.2–67.8) ^1,3^	11.4 (6.2–33.2) ^1,2^	**0.001**
SHs groups, mmol/L	1.86 (1.60–1.97)	1.67 (1.52–1.97) ^2^	1.94 (1.74–2.08) ^1^	1.86 (1.66–1.92) ^1^	**0.011**
SHe groups, mmol/L	19.21 (10.27–25.28)	19.85 (10.5–23.1)	21.91 (11.19–26.06)	16.99 (9.99–23.97)	0.435
Peroxide resistance, %	86.7 (78.7–88.8)	90.6 (58.9–91.8) ^2^	78.7 (73.2–86.1) ^1,3^	86.7 (83.9–87.8) ^2^	**0.001**
Peroxide-induced hemolysis, %	13.3 (112–21.3)	9.4 (8.7–41.1) ^2,3^	21.30 (13.90–26.80) ^1,2^	13.30 (12.25–16.15) ^1,2^	**0.001**
Serum ceruloplasmin, g/L	0.19 (0.11–0.25)	0.19 (0.11–0.26)	0.20 (0.16–0.27) ^3^	0.17 (0.11–0.21) ^2^	**0.015**

Note: Dialysate OS markers (MDAd and TPAd) were available in a subset of patients (n = 48; LCI T1 n = 12, T2 n = 18, T3 n = 18). Data are presented as median (interquartile range, Q25–Q75). Superscript numbers (^1–3^) indicate statistically significant pairwise differences between LCI tertiles based on Dunn’s post hoc test with correction for multiple comparisons: ^1^ significant vs. LCI T1; ^2^ significant vs. LCI T2; ^3^ significant vs. LCI T3. Abbreviations: LCI, lipoprotein combine index; MDAd, dialysate malondialdehyde; MDAe, erythrocyte malondialdehyde; MDAs, serum malondialdehyde; MDAu, urinary malondialdehyde; SHe groups, erythrocyte sulfhydryl groups; SHs groups, serum sulfhydryl groups; TPAd, total peroxidase activity in dialysate; TPAe, total peroxidase activity in erythrocytes.

**Table 3:** Multivariable Linear Regression Models for Dialysate OS Markers.

Predictors	logMDAd Estimate β (95% CI)	*p*-Value	logTPAd Estimate β (95% CI)	*p*-Value
Intercept	3.25 (3.04 to 3.46)	<0.001	3.11 (2.83; 3.38)	<0.001
Sex (male vs. female)	0.35 (−0.22; 0.93)	0.217	1.57 (0.91; 2.24)	<0.001
Diabetes status (yes vs. no)	0.83 (1.36; 0.31)	0.003	−0.67 (−1.27; −0.06)	0.031
Estimated glucose load	0.04 (0.05; 0.025)	<0.001	−0.03 (−0.04; −0.01)	<0.001
Age (years)	0.02 (−0.004; 0.044)	0.098	−0.02 (−0.05; 0.006)	0.123
LogLCI	0.48 (0.29; 0.68)	<0.001	−0.37 (−0.62; −0.12)	0.005

Abbreviations: LogLCI, log-transformed lipoprotein combine index; logMDAd, log-transformed dialysate malondialdehyde; logTPAd, log-transformed total peroxidase activity in dialysate.

## Data Availability

The data presented in this study are available on request from the corresponding author due to ethical restrictions.

## Supplementary Materials

The following supporting information can be downloaded at: https://www.mdpi.com/article/10.3390/biomedicines14020456/s1, Table S1: Fully Adjusted Multivariable Model for logMDAd.; Table S2: Fully Adjusted Multivariable Linear Regression Model for TPAd.

## Abbreviations

The following abbreviations are used in this manuscript:

ACE	Angiotensin-converting enzyme
AIP	Atherogenic index of plasma
BMI	Body mass index
CAPD	Continuous ambulatory peritoneal dialysis
CrCl	Creatinine clearance
CRP	C-reactive protein
D/P Cr	Dialysate-to-plasma creatinine ratio
Hb	Hemoglobin
HDL-C	High-density lipoprotein cholesterol
iPTH	Intact parathyroid hormone
LCI	Lipoprotein combine index
LDL-C	Low-density lipoprotein cholesterol
MDAd	Dialysate malondialdehyde
MDAe	Erythrocyte malondialdehyde
MDAs	Serum malondialdehyde
MDAu	Urinary malondialdehyde
OS	Oxidative stress
PD	Peritoneal dialysis
PET	Peritoneal equilibration test
RAAS	Renin–angiotensin–aldosterone system
RKF	Residual kidney function
SHs	Serum sulfhydryl groups
SHe	Erythrocyte sulfhydryl groups
TC	Total cholesterol
TGs	Triglycerides
TPAd	Total peroxidase activity in dialysate
TPAe	Total peroxidase activity in erythrocytes
