# Supplementary material for: Lipoprotein Combine Index Is Associated with Multi-Compartment Oxidative Stress in Clinically Stable Peritoneal Dialysis Patients: A Cross-Sectional Study
Source: Biomedicines. 2026 Feb 18;14(2):456. doi: 10.3390/biomedicines14020456 (PMC12938693; doi:10.3390/biomedicines14020456)
Supplement: Supplementary file 1 [file biomedicines-14-00456-s001.zip › biomedicines-4154380-supplementary.pdf]

Supplementary File

N. Stepanova, L. Korol. **Lipoprotein Combine Index is Associated with Multi-Compartment Oxidative Stress in Peritoneal Dialysis Patients: A Cross-Sectional Study**

Supplementary Table S1. Fully Adjusted Multivariable Model for logMDAd.

Predictor	Estimate β (95% CI)	p-value
Intercept	3.291 (3.072 to 3.509)	<0.001
Sex (male vs female)	0.952 (0.164 to 1.741)	0.020
Diabetes status (yes vs no)	0.757 (1.348 to 0.166)	0.014
Estimated glucose load (g/d)	0.045 (0.059 to 0.032)	<0.001
Age (years)	0.005 (−0.025 to 0.036)	0.715
LogLCI	0.445 (0.199 to 0.692)	<0.001
Statin therapy (yes vs no)	−0.057 (−0.104 to −0.010)	0.019
Serum albumin (g/L)	0.451 (−0.127 to 1.030)	0.122
logCRP (mg/L)	−0.299 (−0.682 to 0.084)	0.122

Model statistics: $R^2 = 0.689$; Adjusted $R^2 = 0.608$; $F(8,31) = 8.57$; $p < 0.001$

Supplementary Table S2. Fully Adjusted Multivariable Linear Regression Model for TPAd.

Predictor	Estimate β (95% CI)	p-value
Intercept	2.740 (2.443 to 3.038)	<0.001
Sex (male vs female)	0.937 (0.171 to 1.702)	0.017
Diabetes status (yes vs no)	0.279 (−0.566 to 1.124)	0.512
Estimated glucose load (g/d)	−0.017 (−0.031 to −0.004)	0.014
Age (years)	−0.006 (−0.032 to 0.021)	0.672
LogLCI	−0.447 (−0.698 to −0.195)	<0.001
Statin therapy (yes vs no)	0.149 (0.086 to 0.212)	<0.001
Serum albumin (g/L)	0.373 (−0.482 to 1.228)	0.386
logCRP (mg/L)	0.288 (−0.288 to 0.865)	0.321

Model statistics: $R^2 = 0.606$; Adjusted $R^2 = 0.534$; $F(11,60) = 8.40$; $p < 0.001$

Abbreviations: β, regression coefficient; CI, confidence interval; CRP, C-reactive protein; D/P Cr, dialysate-to-plasma creatinine ratio; LCI, lipid composite index; MDAd, malondialdehyde; TPAd, total peroxidase activity; log, natural logarithmic transformation.
